# Involvement of *hcp2* on maintaining cell wall integrity and pathogenicity of *Vibrio alginolyticus* by modulating Sec pathway

**DOI:** 10.1042/BCJ20253168

**Published:** 2025-12-05

**Authors:** Shuilong Wu, Huapu Chen, Yu Huang, Bei Wang, Kwaku Amoah, Jia Cai, Jichang Jian

**Affiliations:** 1 Zhanjiang Institute of Clinical Medicine, Central People’s Hospital of Zhanjiang, Zhanjiang, 524045, China; 2 Guangdong Provincial Key Laboratory of Aquatic Animal Disease Control and Healthy Culture, College of Fishery, Guangdong Ocean University; Key Laboratory of Diseases Controlling for Aquatic Economic Animals of Guangdong Higher Education Institutions, College of Fishery, Guangdong Ocean University, Zhanjiang, 524088, China; 3 Shenzhen Institute of Guangdong Ocean University, Shenzhen, 518120, China

**Keywords:** cAMP–CRP, hcp2, LPS, PGN, Sec pathway

## Abstract

Hemolysin co-regulated protein 2 (Hcp2) is a core component of the type VI secretion system 2 in *Vibrio alginolyticus*, a widespread marine pathogen that infects humans and aquaculture species. Deletion of *hcp2* (Δ*hcp2*) significantly attenuated virulence in zebrafish larvae, showing reduced abdominal edema and impaired toll-like receptor (*tlr*)2 and *tlr4*-mediated innate immune activation compared with the wildtype strain. Enzyme-linked immunosorbent assay and cell lysis assays revealed that the Δ*hcp2* strain exhibited decreased levels of lipopolysaccharide (LPS) and peptidoglycan (PGN), along with increased cell permeability and abnormal cell wall structures observed by transmission electron microscopy. Proteomic and transcriptional analyses further demonstrated that expression of the Sec system components SecB, SecD, and SecF was markedly reduced in the Δ*hcp2* strain and positively regulated by *hcp2*. Bioinformatic prediction combined with protein–DNA docking analysis suggested that the transcription of *secB*, *secD*, and *secF* was co-regulated by the cAMP and cAMP Receptor Protein (cAMP–CRP) complex and RNA polymerase sigma D factor (RpoD), although *rpoD* expression itself remained unaffected. Together with previous evidence that Hcp2 positively regulates cAMP–CRP, these findings suggested that Hcp2 modulated LPS and PGN translocation, probably through the cAMP–CRP pathway, thereby maintaining cell wall integrity and virulence in *V.alginolyticus.*

## Introduction

Type VI secretion system (T6SS) is a versatile and sophisticated bacterial secretion machine that provides multiple benefits to the bacteria that possess it [1,2]. In addition to being a hallmark component of the T6SS injection apparatus, hemolysin co-regulated protein (Hcp) also serves as a receptor and a chaperone that mediates the export of T6SS effectors and regulates T6SS expression through protein–protein interactions [3,4]. However, the intracellular function of Hcp, particularly in processes like regulating cell wall integrity, has received little attention.

The bacterial general secretory (Sec) pathway is a conserved protein translocation system in bacterial and eukaryotic organelles, mediating the transport of newly synthesized proteins (typically in an unfolded or partially folded state) across the cytoplasmic membrane to the periplasm, cell membrane, or endoplasmic reticulum lumen [5,6]. By participating in bacterial cell wall biogenesis and the trans-inner membrane export of pre-effectors from certain bacterial secretion systems, the Sec system plays an essential role in maintaining cell wall integrity and virulence of bacteria [7]. Its core components include SecA, SecYEG, SecDF, SRP, SecB, and YidC. Among that, SecDF is a highly conserved membrane-integrated protein in Gram-negative bacteria and typically exists as a heterodimer localizing in the bacterial inner membrane, physically couples with the SecYEG translocation channel and SecA ATPase, and assists SecA in the post-translocational pushing of substrate proteins, especially the secretion of large or complex structural proteins [8,9]. Previous studies have revealed that SecD/F deletion impairs bacterial virulence factor secretion, disrupts cell wall biogenesis, and increases membrane permeability [10,11]. As the synthesis of unfolded polypeptides progresses, SecB, acting as a universal molecular chaperone of the Sec system, utilizes its potent anti-folding activity to recognize and bind proteins in non-native states, thereby delivering them to SecA in an unfolded state to ensure the proper export of precursor proteins [12,13].

Besides the Sec system, lipopolysaccharide (LPS) and peptidoglycan (PGN) are crucial for maintaining cell wall dynamic equilibrium and structure in bacteria. There are reports demonstrating that the LPS transport (Lpt) system is responsible for the transmembrane transport of LPS [14,15]. Moreover, several components of the Lpt system secretion rely on the Sec pathway, such as LptA–F, which need to be transported to the periplasmic space or the outer side of the inner membrane via the Sec protein secretion system to perform their functions [16,17]. Specifically, the LptD–LptE membrane protein complex is localized in the bacterial outer membrane and is responsible for the final step of LPS transport and assembly [18,19]. Similar to the transmembrane transport of LPS, enzymes and precursor molecules involved in PGN synthesis also need to be transported to the cell wall synthesis site via the Sec pathway to facilitate the repair and expansion of the cell wall. For example, the endolytic PGN transglycosylase rare lipoprotein A (RlpA) can degrade PGN, aiding in the remodeling of the bacterial cell wall during cell division by interacting with other proteins [20–22]. The PGN-associated lipoprotein (Pal) is an outer membrane lipoprotein composed of a signal peptide, a lipid-modified region, and a mature protein, which contributes to the invasion and virulence of pathogenic bacteria [23–25]. Functionally, Pal stabilizes the outer membrane by non-covalently binding to the PGN layer through its periplasmic domain, and this binding is crucial for maintaining the integrity of the cell wall and bacterial survival and pathogenesis [26–28]. Actually, the Sec pathway is crucial for the biogenesis and assembly of LPS and PGN in bacteria [29,30].

Even though evidence from several key studies has demonstrated a close relationship between T6SS and LPS in bacteria [31,32], the intrinsic regulatory role of T6SS on LPS/PGN, both being crucial for bacterial pathogenicity and defense, remains poorly understood. In the study, we compared the LPS and PGN content, cell permeability, and cell wall ultrastructure between wildtype and *hcp2* mutant strains of *Vibrio alginolyticus*. The results indicated that *hcp2* played a significant role in maintaining cell wall integrity. Proteomic analyses revealed that *hcp2* did not affect the expression levels of proteins involved in LPS and PGN biosynthesis and assembly. However, signal peptide prediction results suggested that the transmembrane secretion of several key proteins might rely on the Sec system. Further proteomic and quantitative reverse transcription polymerase chain reaction (qRT-PCR) analyses confirmed that *hcp2* could positively regulate the expression levels of *secB* and *secD/secF*. Moreover, bioinformatic and protein–DNA molecular docking analyses revealed that the cAMP–CRP complex, which was previously shown to be positively regulated by Hcp2, might play a key role in the Hcp2-mediated transcriptional activation of the *secB* and *secD/secF* genes in *V. alginolyticus*. In summary, the study provided experimental evidence and new insights into the regulatory mechanism by which *hcp2* maintained cell wall integrity and virulence of *V. alginolyticus*.

## Results

### Influence of *hcp2* on the pathogenicity and activation ability of inflammatory cytokine in zebrafish

**Figure 1:**
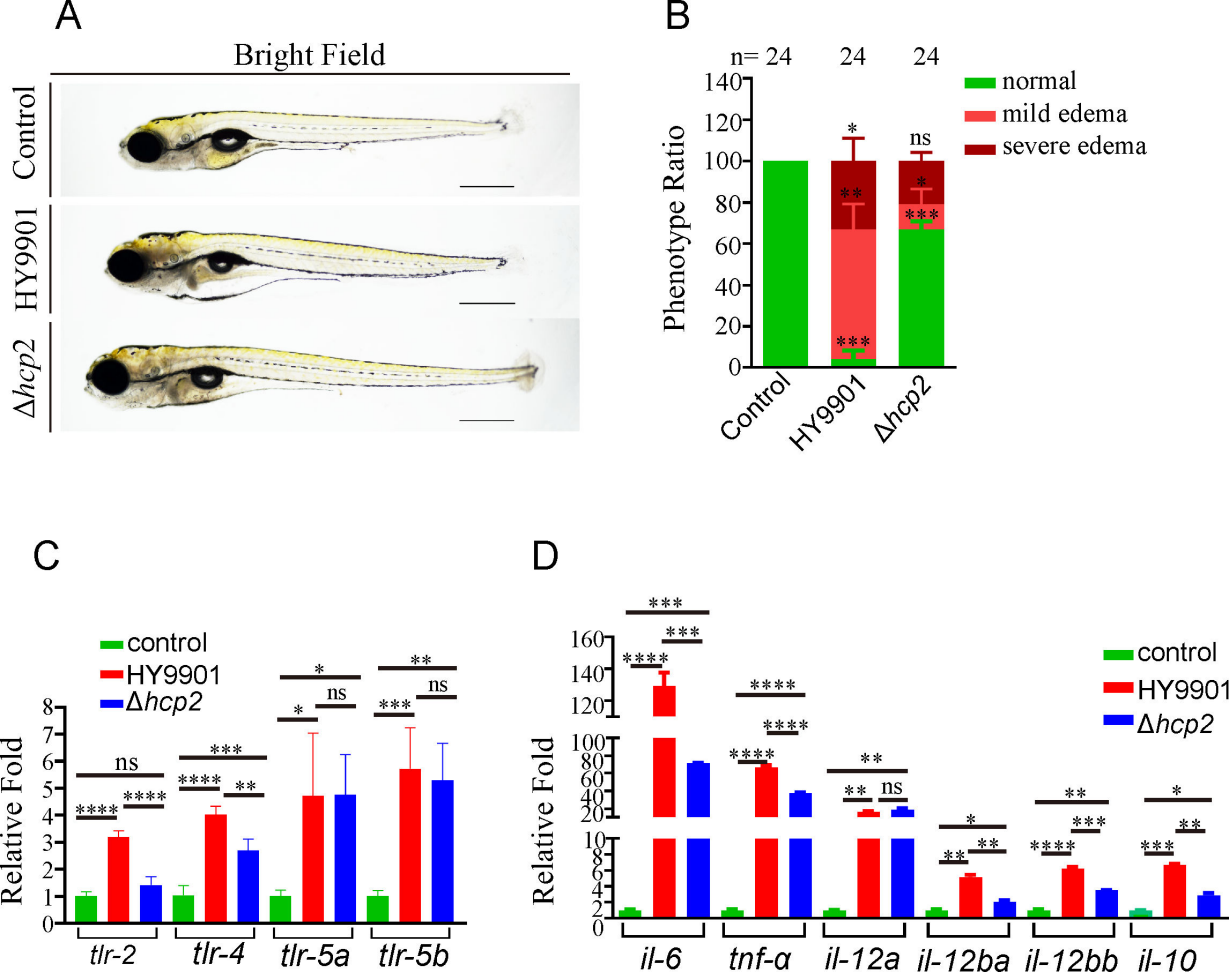
Comparison of the effects of wildtype and *hcp2* mutant strains on virulence and inflammatory activation in zebrafish larvae. (**A-B**) Phenotypes and their ratios of zebrafish larvae infected with wildtype *V. alginolyticus* and *hcp2* mutant strains. Greater transparency in the ventral region of the yolk sac and intestine was indicative of more severe edema in zebrafish. (**C**) Transcriptional levels of innate immune receptors *tlr2*, *tlr4*, and *tlr5* in zebrafish infected with the wildtype and the *hcp2* mutant strains. (**D**) Transcriptional analysis of inflammatory factors *il6*, *il10*, *il12*, and *tnf-α* in zebrafish infected with wildtype and *hcp2* mutant strains. Scale bars: 500 μm in Figure 1A, n: number of analyzed zebrafish larvae, *P < 0.05, **P < 0.01, ***P < 0.001, ****P < 0.0001. ns, no significance; *tnf-α*, tumor necrosis factor*-α*.

Our previous studies have demonstrated that the deletion of *hcp2* attenuates the *V. alginolyticus* pathogenicity toward zebrafish, as evidenced by lower mortality ratios in both larvae and adult zebrafish infected with the *Δhcp2* strain compared with those infected with the wildtype strain [33]. Additionally, we observed the live larval phenotypes after the challenge experiment. The results showed attenuated virulence, characterized by a lower proportion of mild and severe edema phenotypes and a higher proportion of edema-free individuals in the *Δhcp2* strain infection group compared with the wildtype strain infection group (Figure 1A and B). Further analysis of the inflammatory response via qRT-PCR revealed that the transcriptional levels of toll-like receptor (*tlr*)*2* and *tlr4* in zebrafish infected with the wildtype strain HY9901 were significantly higher than those in the *Δhcp2* strain-infected group, except for the transcriptional levels of *tlr5* (two paralogs *tlr5a and tlr5b* in zebrafish), which showed no significant differences between the wildtype and *hcp2* mutant-infected groups, although they were significantly elevated compared with the non-infected group (Figure 1C). Simultaneously, the transcriptional levels of pro-inflammatory cytokines interleukin (*il*)-6/*il*10/*il*12, and tumor necrosis factor*-α* in the HY9901 infected group were significantly higher than in the *Δhcp2* strain infected group (Figure 1D).

### Effects of the *hcp2* mutation on lipopolysaccharide, peptidoglycan content, and cell wall ultrastructure of *V. alginolyticus*

**Figure 2:**
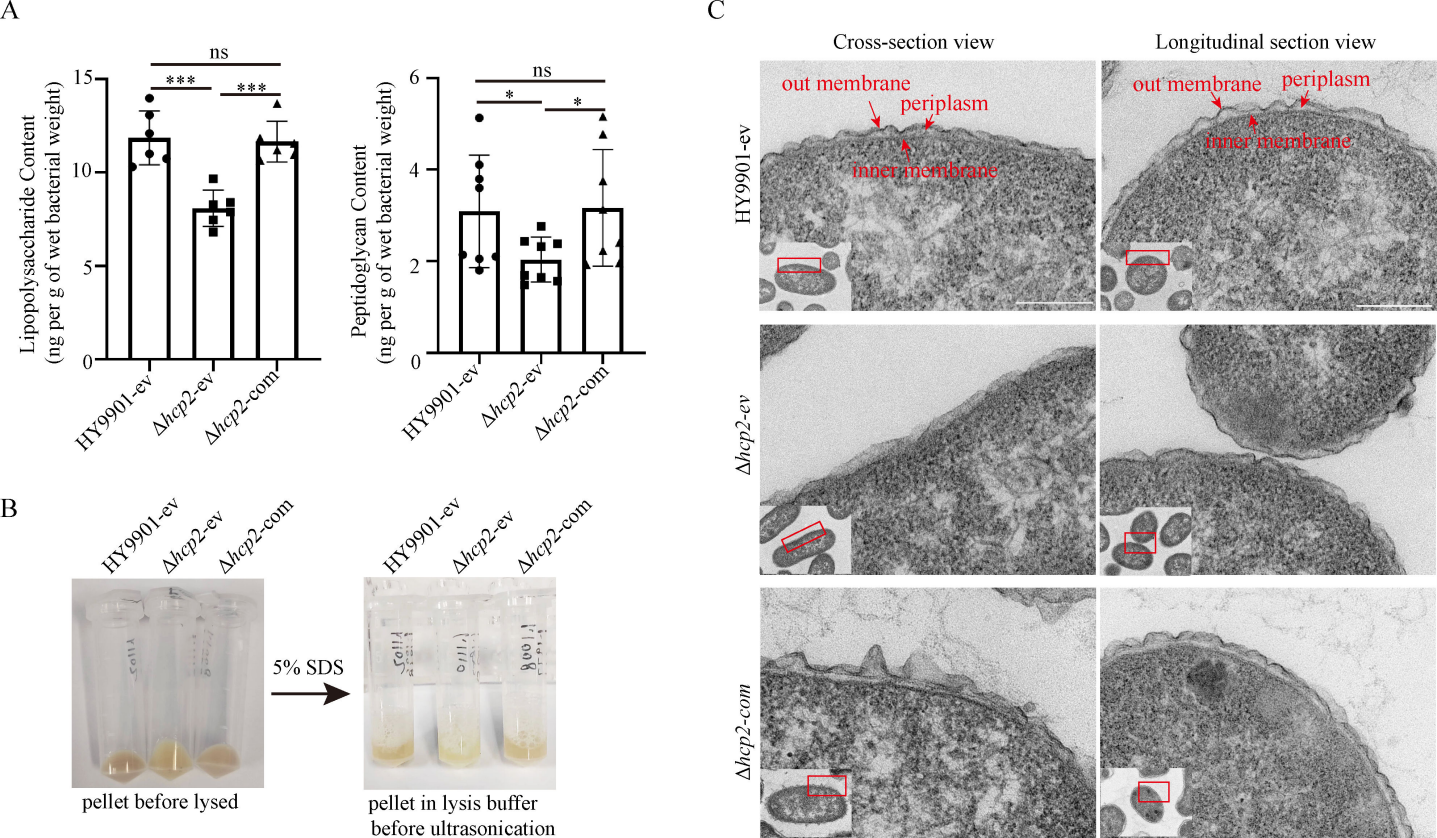
Quantitative analysis of LPS and PGN and ultrastructural observation of the cell wall of *V. alginolyticus.* (A) Quantitative analysis of LPS and PGN in *V. alginolyticus* HY9901, *hcp2* mutant, and *hcp2* complement strains. (**B**) The pellet state and the solubility of *V. alginolyticus* HY9901-ev, Δ*hcp2*-ev, and Δ*hcp2*-com strains in 5% SDS lysis buffer before ultrasonication. The mutant strain displayed a phenotype of increased susceptibility to lysis, yielding a lysate that was notably more transparent compared with the wildtype and complemented strains. (**C**) Comparison of cell wall ultrastructure in *V. alginolyticus* HY9901-ev, Δ*hcp2*-ev, and Δ*hcp2*-com strains. The TEM results showed that the *hcp2* mutant strain had a lower electron density of the outer membrane and a more blurred boundary with the periplasm compared with wildtype strain. The phenotype defect caused by the loss of *hcp2* was partially rescued in the complemented strain. HY9901-ev: the wildtype strain carried with pBBR1-MCS1 empty vector; Δ*hcp2-*ev: the Δ*hcp2* strain carried with pBBR1-MCS1 empty vector; Δ*hcp2-*com: the Δ*hcp2* strain carried with pBBR1-MCS1-*hcp2* vector. *P < 0.05, ***P < 0.001. ns, no significance.

In mammals and teleosts, *tlr2* serves as the primary receptor for recognizing and binding to PGN, and though the sensitivity of *tlr4* in teleosts to LPS released by pathogens is low, it also plays an important role in activating the host’s innate immune response [34,35]. To determine whether the reduced expression of *tlr2* and *tlr4* following infection of zebrafish with the *Δhcp2* strain is associated with PGN and LPS, we quantitatively analyzed the LPS and PGN content of *V. alginolyticus*. The results showed that compared with the wildtype strain HY9901, the LPS and PGN content of the *Δhcp2* strain was significantly reduced and could be recovered in the *hcp2* complement strain (Figure 2A). Additionally, the mutant strain appeared more susceptible to lysis by 5% SDS, as evidenced by the formation of a clearer lysate, whereas the lysate of the complemented strain was comparable to that of the wildtype strain (Figure 2B). Subsequently, transmission electron microscopy (TEM) revealed that the loss of *hcp2* resulted in abnormal cell wall structure of *V. alginolyticus*, characterized by blurred structures of the cytoplasm, periplasmic space, and inner membrane, as well as decreased electron density of the outer membrane. Moreover, the defect phenotype could be partially restored, as evidenced by distinct cell wall boundaries and a restored electron density of the outer membrane in the complemented strain (Figure 2C). The results demonstrated that *hcp2* was essential for the cell envelope integrity of *V. alginolyticus*.

### Expression analysis of proteins related to LPS and PGN biosynthesis and transport based on omics data and prediction of their signal peptides

To investigate the reasons for the reduced content of LPS and PGN in the Δ*hcp2* strain of *V. alginolyticus*, we analyzed the relative expression levels of proteins involved in LPS and PGN synthesis, transport, and assembly based on the mut/wt value from omics data. Additionally, we predicted the signal peptides in these proteins. The results showed that, compared with the wildtype strain, there were no significant differences in the expression levels of proteins related to LPS and PGN synthesis and transport in the Δ*hcp2* strain. However, further bioinformatic prediction showed that several proteins involved in LPS export, such as LptA, LptC, LptD, and LptE, possess signal peptides dependent on the Sec/SPI or Sec/SPII secretion systems. Similarly, proteins related to PGN synthesis, such as RlpA and Pal, also contain signal peptides dependent on the Sec/SPII secretion system (Table 1 and Supplementary Figure S1). The prediction indicated that the transmembrane export of LPS and PGN might occur via the Sec pathway in *V. alginolyticus*.

**Table 1:** Proteomic analysis of proteins involved in LPS and PGN synthesis and assembly, and prediction of their signal peptides

Gene name	Protein description	mut/wt	Signal peptide	Likelihood
*lptD*	LPS-assembly protein LptD	1.086	Protein type signal peptide (Sec/SPI)	0.8616
*lptE*	LPS-assembly lipoprotein LptE	0.924	Lipoprotein signal peptide (Sec/SPII)	0.9982
*rfaF*	ADP-heptose--LPS heptosyltransferase II	0.899	None	< 0.2
*clpS*	ATP-dependent Clp protease adapter protein ClpS	1.265	None	< 0.2
*msbA*	Transport ATP-binding protein MsbA	1.058	None	< 0.2
*VMC_14400*	Putative lipopolysaccharide A protein	1.264	None	< 0.2
*lptC*	Lipopolysaccharide export system protein LptC	1.194	Lipoprotein signal peptide (Sec/SPII)	0.2908
*VMC_34720*	Lipopolysaccharide export system ATP-binding protein LptB	1.134	None	< 0.2
*VMC_14430*	Lipopolysaccharide biosynthesis protein	1.089	None	< 0.2
*lapB*	Lipopolysaccharide assembly protein B	1.083	None	0.2103
*lptA*	Lipopolysaccharide export system protein LptA	1.047	Protein type signal peptide (Sec/SPI)	0.9959
*VMC_18720*	Lipopolysaccharide export system permease protein LptF	1.016	None	< 0.2
*mrdA*	Peptidoglycan D, D-transpeptidase MrdA	1.227	None	< 0.2
*mrdB*	Peptidoglycan glycosyltransferase MrdB	1.198	None	< 0.2
*rlpA*	Endolytic peptidoglycan transglycosylase RlpA	N/A	Lipoprotein signal peptide (Sec/SPII)	0.9995
*ftsW*	Probable peptidoglycan glycosyltransferase FtsW	N/A	None	< 0.2
*ftsI*	Peptidoglycan D, D-transpeptidase FtsI	1.098	None	< 0.2
*pal*	Peptidoglycan-associated protein	0.991	Lipoprotein signal peptide (Sec/SPII)	0.9985
*htrB*	Lipid A biosynthesis lauroyltransferase	1.131	None	< 0.2
*lpxM*	Lipid A biosynthesis myristoyltransferase	1.148	None	< 0.2
*lpxB*	Lipid-A-disaccharide synthase	1.16	None	< 0.2
*mviN*	Probable lipid II flippase MurJ	N/A	None	0.21
*VMC_32580*	Cyclopropane-fatty-acyl-phospholipid synthase	1.058	None	< 0.2
*VMC_33470*	Probable lipid kinase YegS-like	0.941	None	< 0.2
*VMC_33830*	Putative phospholipid biosynthesis acyltransferase	1.339	None	< 0.2
*VMC_34660*	Intermembrane phospholipid transport system permease protein MlaE	1.083	None	< 0.2

Notes: Protein type signal peptide (Sec/SPI) refers to the general protein secretory pathway widely utilized in bacteria. Lipoprotein signal peptide (Sec/SPII) refers to a signal peptide directing proteins to the periplasm via the Sec translocon, where it is cleaved by signal peptidase II and subsequently lipidated, ultimately yielding a functional membrane-anchored lipoprotein. mut: *hcp2* mutant*,* wt: wildtype strain HY9901. Likelihood: The probability that the sequence belongs to a specific category of signal peptides. N/A: Data not shown due to either sequencing failure or value below the detection limit.

### Analysis of expression levels of genes related to the Sec system

**Figure 3:**
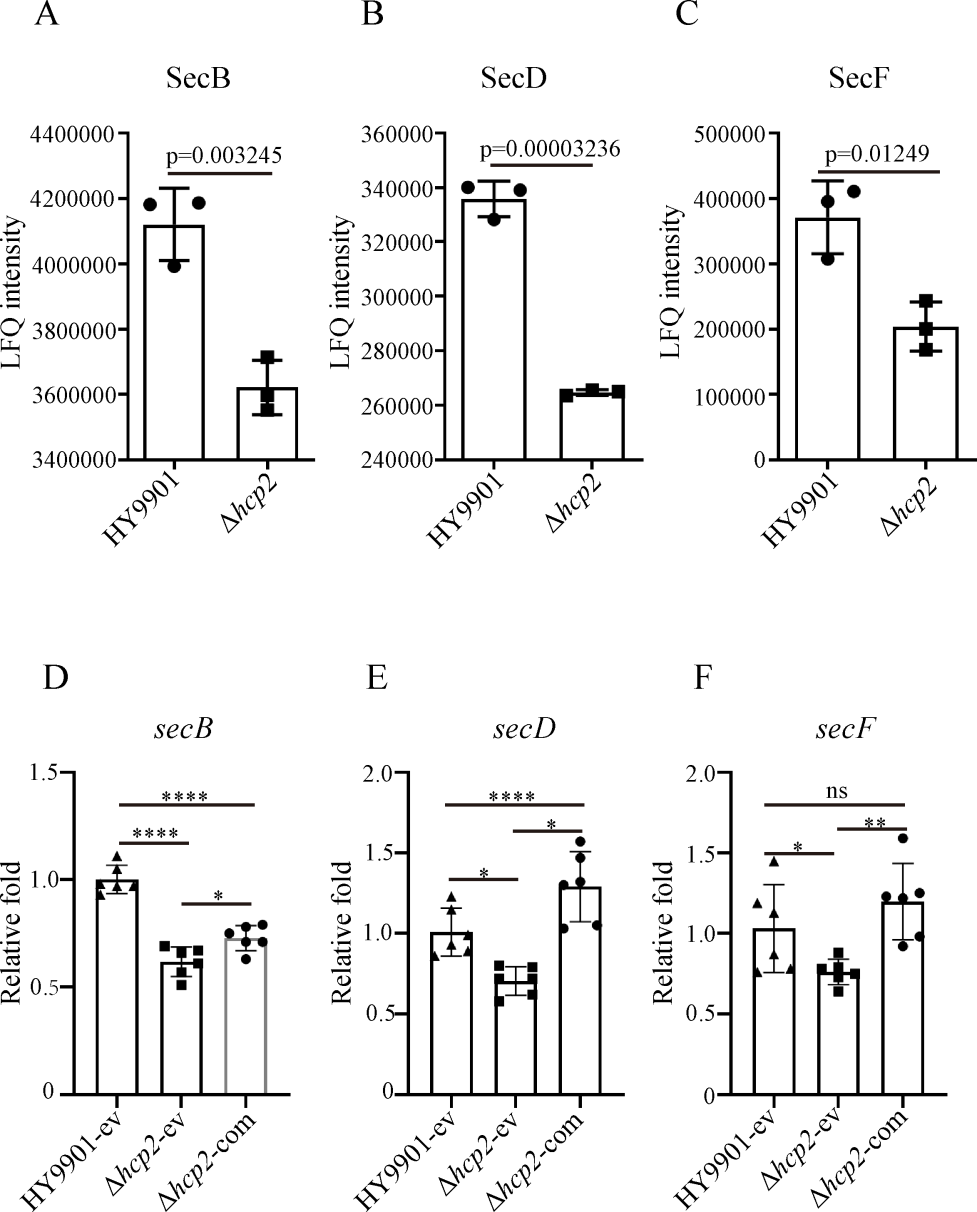
Effect of *hcp2* on the expression of particular components of the Sec system in *V. alginolyticus.* (**A-C**) Relative quantitative analysis of SecB, SecD, and SecF proteins in the wildtype strain and the *hcp2* mutant strain; (**D-F**) Transcriptional level analysis of *secB*, *secD*, and *secF* genes in the wildtype strain, *hcp2* mutant strain, and its complemented strain. HY9901-ev: the wildtype strain carried with pBBR1-MCS1 empty vector; Δ*hcp2-*ev: the Δ*hcp2* strain carried with pBBR1-MCS1 empty vector; Δ*hcp2-*com: the Δ*hcp2* strain carried with pBBR1-MCS1-*hcp2* vector. *P < 0.05, **P < 0.01, ****P < 0.0001. ns, no significance.

The proteomics results showed that the absence of *hcp2* does not affect the expression levels of proteins related to the synthesis and assembly of LPS and PGN, but the predicted signal peptides of LptA/C/D/E and RlpA/Pal raised the possibility that their export from cytoplasm might be mediated by the Sec system. Therefore, we further analyzed whether the Sec proteins were affected in the *hcp2* mutant. Proteomic analysis revealed that the protein abundances of SecB, SecD, and SecF were markedly decreased in the Δ*hcp2* strain relative to the wildtype strain (Figure 3A–3C). Consistently, qRT-PCR results demonstrated that the transcription of *secB*, *secD*, and *secF* was positively regulated by *hcp2* (Figure 3D–3F).

### Bioinformatic analysis of *sec* gene regulation and the molecular role of Hcp2 in LPS and PGN assembly

**Figure 4:**
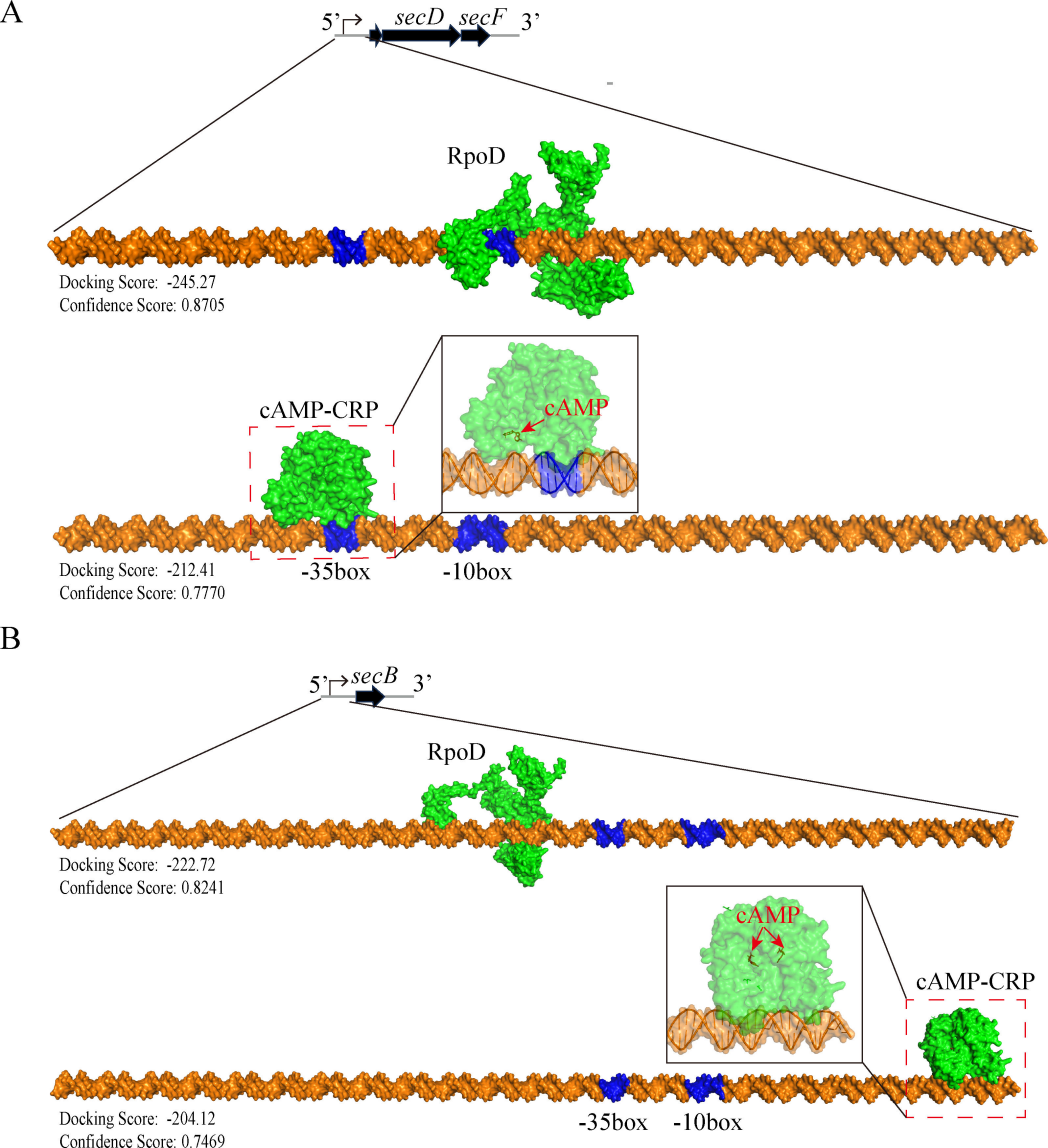
Identification of promoter regions of *secB* and *secD/secF* and binding sites for RpoD and the cAMP–CRP complex based on bioinformatic analysis. (A) Predicted promoter regions and DNA binding sites of RpoD and cAMP–CRP complex in the upstream sequence of *secD*/*secF* genes. (**B**) Predicted promoter regions and DNA binding sites of RpoD and cAMP–CRP complex in the upstream sequence of *secB* gene.

**Figure 5:**
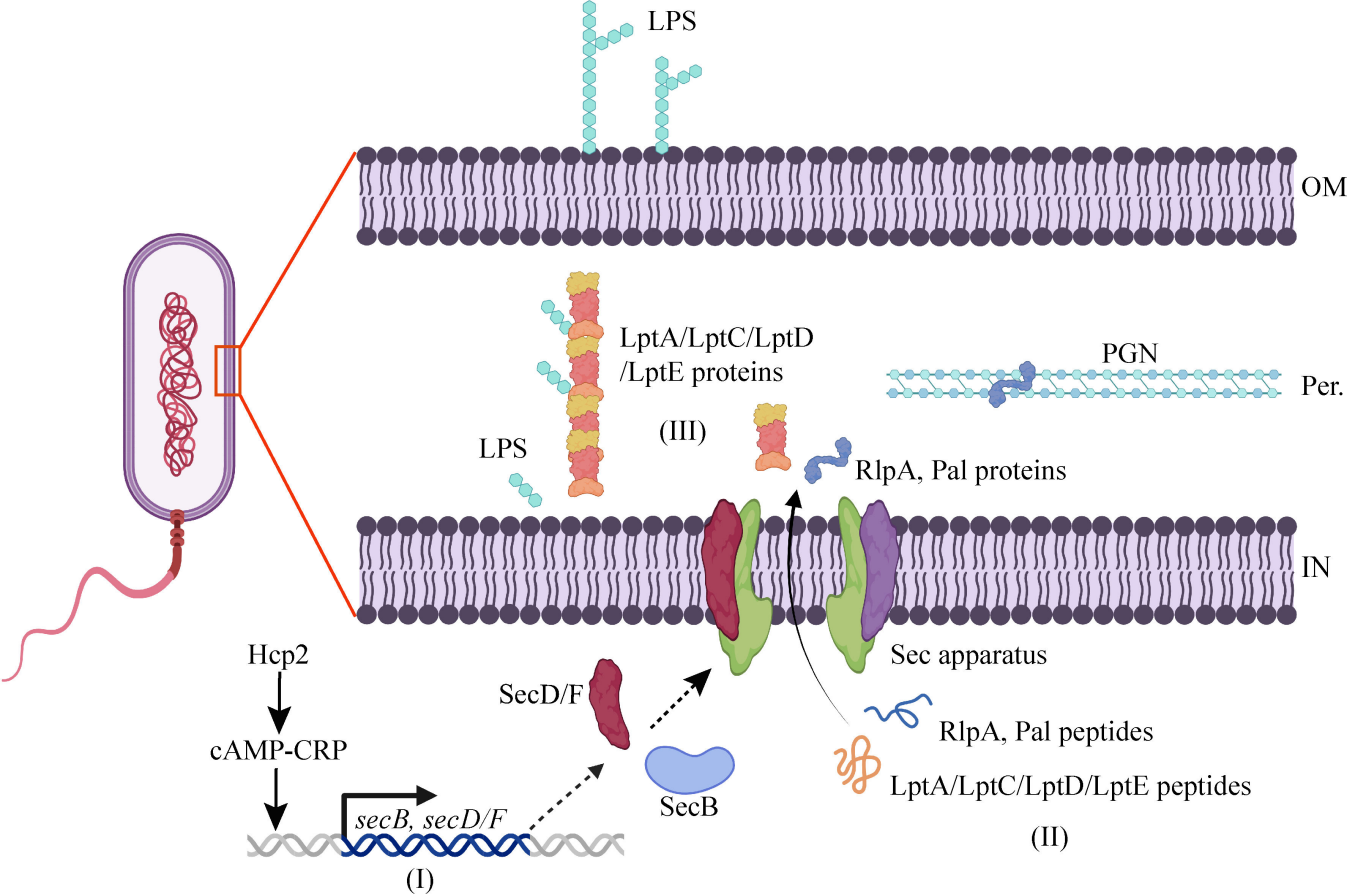
Proposed mechanism by which Hcp2 regulated LPS and PGN assembly in *V. alginolyticus.* This regulatory process was achieved through three key steps: (**I**) Hcp2 modulates the expression of *secB* and *secD/F* via the cAMP–CRP pathway. (**II**) Hcp2 positively influenced the trans-membrane secretion of LPS, PGN assembly associated proteins. (**III**) Consequently, the extraction of LPS and the remodeling of PGN were affected by Hcp2. Black solid arrows represent positive regulatory effects, as supported by our previous findings and the current study. Black dashed arrows denote the translation and inner membrane localization processes of these genes. The red square and line highlight a magnified view of the cell wall structure (left). OM, outer membrane; IN, inner membrane; Per., periplasm; LPS, lipopolysaccharide; PGN, peptidoglycan.

Previous research has shown that Hcp1 and Hcp2 in *V. alginolyticus* are extensively involved in the metabolism of bacterial substrates and biofilm formation, with mechanisms related to the synergistic regulation of intracellular cAMP–CRP complex levels by Hcp1/Hcp2 [36]. In order to elucidate the molecular mechanisms by which *hcp2* positively regulates *secB*, *secD*, and *secF* expression, we conducted promoter prediction and protein–DNA molecular docking analysis on the upstream sequences of the transcription start sites of these genes using specialized software. The results indicated that the upstream regions of these genes harbored promoters that were specifically recognized and activated by the transcription factor RpoD (σ⁷⁰), and binding sites for the cAMP–CRP complex were also found around the promoters (Figure 4A and B and Supplementary Figure S2A-2D). The proteomics sequencing indicated a slight decrease in RpoD expression levels in the Δ*hcp2* strain compared with the wildtype strain, with a ratio of mut/wt at 0.924 and a *P* value of 0.001671. However, the absolute value of the fold change was less than 1.1, suggesting that this difference is not substantial (Supplementary Figure S2E). Simultaneously, the transcription level of *rpoD* was not significantly regulated by *hcp2*, as shown in Supplementary Figure S2F. Based on these findings, we proposed a likely schematic model depicting the role of *hcp2* that the cAMP–CRP complex served as the primary pathway through which *hcp2* regulated *sec* gene expression, thereby modulating LPS and PGN assembly in *V. alginolyticus* (Figure 5).

## Discussion

Pathogens activate innate immunity via *tlr*s that recognize pathogen-associated molecular patterns (PAMPs) and damage-associated molecular patterns (DAMPs). *Tlr*2 primarily detects Gram-positive bacterial components like lipoproteins and lipoteichoic acid in the thick PGN layer and may also recognize specific LPS structures from some Gram-negative bacteria. *Tlr*2 activation recruits MyD88 via its TIR domain, leading to NF-κB and AP-1 activation and inflammatory cytokine production [37]. Although teleost *Tlr*4 does not directly bind LPS like mammalian *tlr*4, it triggers similar downstream MyD88-dependent signaling, activating NF-κB and MAPK pathways to induce inflammatory factors [38,39]. *Tlr*5 recognizes flagellin and initiates pro-inflammatory responses [40]. In this study, zebrafish challenged with *V. alginolyticus* exhibited a significant up-regulation of *tlr5* expression, but no difference was observed between wildtype and *hcp2* mutant strains, despite previous findings that *hcp2* mutation reduced flagellin level. This discrepancy might be explained by (1) possible minimal flagellin secretion during *in vivo* infection, undetectable in transcript analysis; (2) the composition of flagella from over 2000 flagellin subunits, suggesting *hcp2* deletion might not substantially affect overall flagellin expression or antigenicity [41].

Many enzymes and lipoprotein cofactors required for LPS transport/assembly and for PGN synthase regulation are exported across the cytoplasmic membrane by the Sec pathway and are then targeted to the periplasm or outer membrane via the Lol/Lpt machineries [42–44]. The components and functional characteristics of the Sec system are as follows [6]: SecA is an ATPase that provides the energy required for driving protein transmembrane transport. SecYEG is a transmembrane channel complex responsible for protein translocation across the membrane. SecB primarily assists in the binding of unfolded proteins to SecA. Other accessory proteins, such as SecDF and YidC, are involved in protein transport and membrane integration [45]. In the study, although the expression levels of proteins involved in LPS and PGN biosynthesis, such as LptA, LptC, LptD, LptE, RlpA, and Pal, were not affected by the deletion of *hcp2*, the bioinformatics analysis revealed that their transmembrane secretion might rely on the Sec pathway. Moreover, omics data showed that the protein levels of SecB, SecD, and SecF were significantly reduced in the *hcp2* mutant, and their transcription levels were positively regulated by *hcp2*. Therefore, the deletion of *hcp2* might lead to impaired export of LptA/C/D/E, RlpA, and Pal, which would further hinder the proper localization and assembly of PGN and LPS in the periplasm and outer membrane, respectively.

Recent studies indicate that the T6SS rapidly assembles in response to outer membrane stress and extensively interacts with other secretion systems, such as the LPS assembly machinery, through substrate sharing [46,47]. However, the molecular mechanism by which T6SS regulated the processes remains unknown and warrants further investigation. In bacteria, the cAMP–CRP complex acts as a global regulator that binds to specific DNA sites, alters local DNA conformation, and modulates RNA polymerase binding to fine-tune gene transcription [48,49]. RpoD is responsible for recognizing and binding to conserved promoter regions, assisting RNA polymerase in positioning at the promoter region, thereby initiating gene transcription [50,51]. The bioinformatics analysis of the upstream sequences of *secB*, *secD, and secF* genes revealed the presence of promoter sites dependent on the binding of the transcription factor RpoD in *V. alginolyticus*. Moreover, these sites were also found to be adjacent to binding sites for CRP (in the form of the cAMP–CRP complex). Thus, it was hypothesized that the expression of *secB*, *secD*, and *secF* was co-regulated by RpoD and cAMP–CRP complex.

cAMP production depends on the essential interaction between adenylate cyclase (AC) and glucose-specific EIIA (EIIA^glc^), a component of the bacterial phosphotransferase system [52,53]. Our previous study has shown that the role in cAMP production of Hcp2 was potentially mediated through its interaction with EIIA^glc^, leading to a likely synergistic promotion of EIIA^glc^–AC binding in *V. alginolyticus*, which was consistent with the classic molecular chaperone function of Hcp2 [36]. By integrating our experimental data with previous findings, we proposed that Hcp2 might regulate the expression of specific Sec system components, including SecB, SecD, and SecF, through the cAMP–CRP pathway, thereby influencing the transmembrane secretion of proteins involved in the export of LPS and PGN. Therefore, a fully functional T6SS was crucial for maintaining osmotic balance and virulence of *V. alginolyticus* to cope with the ever-changing extracellular milieu.

## Materials and methods

### Bacteria, zebrafish, and culture conditions

*V. alginolyticus* strain HY9901, isolated and identified from diseased red snapper in our laboratory [54], was cultured in TSB medium at 28 ℃ with shaking at 220 rpm. Adult AB zebrafish were purchased from the National Zebrafish Resource Center of China and maintained in the standard zebrafish facility at the Clinical Medical Research Institute of Central People’s Hospital of Zhanjiang. AB zebrafish embryos were obtained by placing male and female zebrafish in a 1:1 ratio in mating tanks one day in advance and collecting the eggs the next morning. Except for experimental requirements, zebrafish embryos were raised according to standard procedures. All zebrafish procedures complied with the ‘Regulations for the Administration of Laboratory Animals in Guangdong Province (2010).’

### Construction of the *hcp2* knockout strain of *V. alginolyticus* and the *hcp2* complement strain

The *hcp2* mutant and complement strains used in this study were constructed and identified as described in our published literature [33]. The knockout strain of the *hcp2* gene in *V. alginolyticus* was constructed using homologous recombination mediated by the recombinant suicide plasmid pLP12-*hcp2*. The complement strain was constructed using the recombinant vector pBBR1-MCS1-*hcp2*.

### Relative quantitative analysis of proteins related to LPS and PGN assembly, Sec system components, and transcription factor RpoD

The whole-cell proteomics sequencing methodology comparing wildtype and *hcp2* mutant strains has been previously described in detail [33]. Building upon these proteomic findings, we conducted a comprehensive analysis of relative protein expression levels associated with the synthesis, transport, and assembly of LPS, PGN, and lipids. Furthermore, we performed comparative quantification of differentially expressed components using label-free quantification, focusing specifically on elements of the Sec system and the transcription factor RpoD.

### Zebrafish larvae infection, imaging, and inflammatory factors detection by qRT-PCR

The overnight culture of *V. alginolyticus* was adjusted to an OD_600_ of 0.15 and then inoculated at a 1:100 ratio into fresh TSB medium. The culture was incubated at 28 ℃ with shaking at 220 rpm until the logarithmic growth phase (∼ 4 h). The cell count of the cultured *V. alginolyticus* suspension was determined using the colony counting method. Subsequently, *V. alginolyticus* wildtype and mutant strains were inoculated at a concentration of 10^8^ CFU per well into six-well plates containing eight 4-days post-fertilization zebrafish larvae that had been pre-grouped and continued to be cultured statically at 28 ℃ for 45 h, with wells containing only an equal number of zebrafish larvae without *V. alginolyticus* serving as the control group. Immediately following the infection, total RNA was extracted from the zebrafish larvae, reverse transcribed into cDNA, and used for qRT-PCR analysis. The methods for total RNA extraction and cDNA preparation from zebrafish larvae were performed as described in the literature [33]. The qPCR primers used in the study were listed in Supplementary Table S1.

For imaging, post-infection, larvae were anesthetized with 200 mg/l tricaine methanesulfonate (MS-222; E10521, Sigma), embedded in 1% low-melting-point agarose (16520100, Invitrogen), and imaged using an upright fluorescence microscope (Ni-E, Nikon, Japan). For euthanasia, larvae were placed in an ice-water mixture to induce hypothermia, consistent with our standard laboratory protocol. Adult zebrafish were killed by immersion in a pharmaceutical-grade MS-222 solution (400 mg/l) for a minimum of 10 min following cessation of opercular movement, as per the NIH Intramural Research Program’s guidelines for Zebrafish Animal Study Proposals.

### Determination of LPS, PGN content

The overnight culture of *V. alginolyticus* was adjusted to an OD_600_ of 0.15, then inoculated at a 1:100 ratio into fresh TSB medium and cultured at 220 rpm, 28 ℃ for 16 h. A 2 ml aliquot of the bacterial suspension was centrifuged at 4500 rpm for 10 min at 4 ℃, and the wet weight of the bacterial pellet was measured. Bacterial LPS and PGN were extracted according to the reports [55,56], with minor modifications. The pellet was washed three times with cold phosphate-buffered saline (PBS) and finally resuspended in 200 µl of PBS containing 5% SDS. The bacterial suspension was sonicated on ice at 150 W for 5 min with a cycle of 1 sec on and 3 sec off. It was then boiled in a metal bath at 100 °C for 2 h. Finally, the sample was centrifuged at 20,000× ***g*** for 10 min at room temperature (RT), and the supernatant was collected. Then, the concentration of LPS and PGN in the extracted supernatant was determined using the bacterial LPS-ELISA Kit (LM8K8340, Shanghai Lianmai Biotechnology) and the bacterial PGN-ELISA Kit (LM8K365, Shanghai Lianmai Biotechnology), respectively. The experimental procedures were carried out according to the instructions provided with the kits.

### Transmission electron microscopy (TEM) analysis of cell wall structure

Overnight-cultured *V. alginolyticus* suspension was collected and centrifuged at 4000x ***g*** for 2 min at RT. The bacterial pellet, equivalent to the size of a mung bean, was gently resuspended in electron microscopy fixative (G1102, Servicebio) and fixed at 4 ℃ for 2–4 h. Cells were centrifuged at 12,000x ***g*** for 5 min, and the pellet was resuspended in 0.1 M PBS (pH 7.4), washed for 3 min, and centrifuged again, repeated three times. Before agarose solidified, the bacterial pellet was suspended within the agarose using tweezers. Samples were post-fixed in 1% osmium tetroxide (Ted Pella Inc, 18456) prepared in 0.1 M phosphate buffer (PB, pH 7.4) for 2 h at RT, avoiding light. The pellet was washed three times with 0.1 M PB (pH 7.4), 15 min each. Tissues were subjected to ascending dehydration in a graded ethanol series (30%, 50%, 70%, 80%, 95%, and 100% ethanol) for 20 min each, followed by two 15 min incubations in 100% acetone. The cells were infiltrated with a mixture of acetone and 812 embedding medium (SPI, 90529-77-4) at a ratio of 1:1 for 2–4 h at 37 ℃, then at a ratio of 1:2 overnight at 37 ℃. The pellet was embedded in pure 812 embedding medium for 5–8 h at 37 ℃. Samples were inserted into embedding molds and incubated at 37 ℃ overnight. Molds were placed in an oven at 60 ℃ for polymerization for 48 h. Polymerized resin blocks were sectioned into ultrathin slices (60–80 nm) using an ultramicrotome (Leica UC7, Leica). Then, the small piece was collected on 150-mesh Formvar-coated copper grids (WFHM-150, Servicebio). Copper grids were stained for 8 min in a saturated ethanol solution of 2% uranyl acetate (SPI, 02624-AB) in the dark, followed by three 70% ethanol washes and three ultrapure water washes. Then, stained for 8 min in a 2.6% lead citrate solution under a carbon dioxide-free environment, followed by three ultrapure water washes. Stained sections were placed in copper grid boxes and left to dry at RT overnight. Images were collected and analyzed using a transmission electron microscope (HITACHI HT7800). The formulation of 2.6% lead citrate solution is as follows: lead nitrate (Sigma, 203580) 1.33 g, sodium citrate (Sinopharm Chemical Reagent Co., Ltd., 10019408) 1.76 g, and double-distilled water 30 ml were mixed by shaking until a milky appearance was observed. Freshly prepared 1 M sodium hydroxide (8 ml) was added, followed by double-distilled water to a final volume of 50 ml. The pH was adjusted to 12.

### Bioinformatics analysis

The promoter sequences and their locations of the *secB* and *secD/F* genes were predicted using the Softberry server (http://www.softberry.com/). Signal peptide sequences of the proteins were predicted using the SignalP website (https://services.healthtech.dtu.dk/service.php?SignalP-5.0). The three-dimensional structures of the RpoD and CRP proteins from *V. alginolyticus* were modeled using the SWISS-MODEL database (https://swissmodel.expasy.org/). Subsequently, protein–DNA molecular docking analysis was performed using the HDOCK server (http://hdock.phys.hust.edu.cn/). The docking results of protein–DNA interactions were visualized and analyzed using PyMOL software.

### Statistical analysis

Data are presented as the mean ± standard error of the mean. All experiments were performed at least in triplicate. Statistical significance was determined using a two-tailed Student’s t-test (for comparisons between two groups) or one-way analysis of variance (ANOVA, for comparisons among three or more groups) with GraphPad Prism 5 software. All data analyses were conducted with a 95% confidence interval. The *P* value of less than 0.05 (*P* <0.05) was considered statistically significant.

## Supplementary material

online supplementary material 1

## Data Availability

The data analyzed in the study are available within the article and its supplementary files or from the corresponding author upon request.

## Open Access

This article has been published open access under our Subscribe to Open programme, made possible through the support of our subscribing institutions, learn more here: https://portlandpress.com/pages/open_access_options_and_prices#conditional

## Abbreviations

AC: adenylate cyclase
AP-1: Activator protein 1
Hcp: hemolysin co-regulated protein
Hcp2: hemolysin co-regulated protein 2
LPS: lipopolysaccharide
Lpt: lipopolysaccharide transport
MAPK: Mitogen-activated protein kinase
MyD88: Myeloid Differentiation Primary Response 88
PB: phosphate buffer
PBS: phosphate-buffered saline
PGN: peptidoglycan
Pal: peptidoglycan-associated lipoprotein
RT: room temperature
RlpA: rare lipoprotein A
SRP: Signal recognition particle
Sec: secretory
TEM: transmission electron microscopy
TIR: Toll / Interleukin-1 receptor homology domain
TSB: Tryptic soy broth
T6SS: type VI secretion system
cDNA: Complementary DNA
clpS: Caseinolytic peptidase adaptor protein S
il: interleukin
lptA: Lipopolysaccharide transport protein A
lptC: Lipopolysaccharide transport protein C
lptD: Lipopolysaccharide transport protein D
lptE: Lipopolysaccharide transport protein E
qPCR: Quantitative polymerase chain reaction
tlr: toll-like receptor
