# Supplementary material for: Involvement of hcp2 on maintaining cell wall integrity and pathogenicity of Vibrio alginolyticus by modulating Sec pathway
Source: Biochem J. 2025 Dec 5;482(23):1779–91. doi: 10.1042/BCJ20253168 (PMC12751085; doi:10.1042/BCJ20253168)
Supplement: online supplementary material 1 [file bcj-482-23-BCJ20253168-s001.docx]

**Supplementary Materials**

**
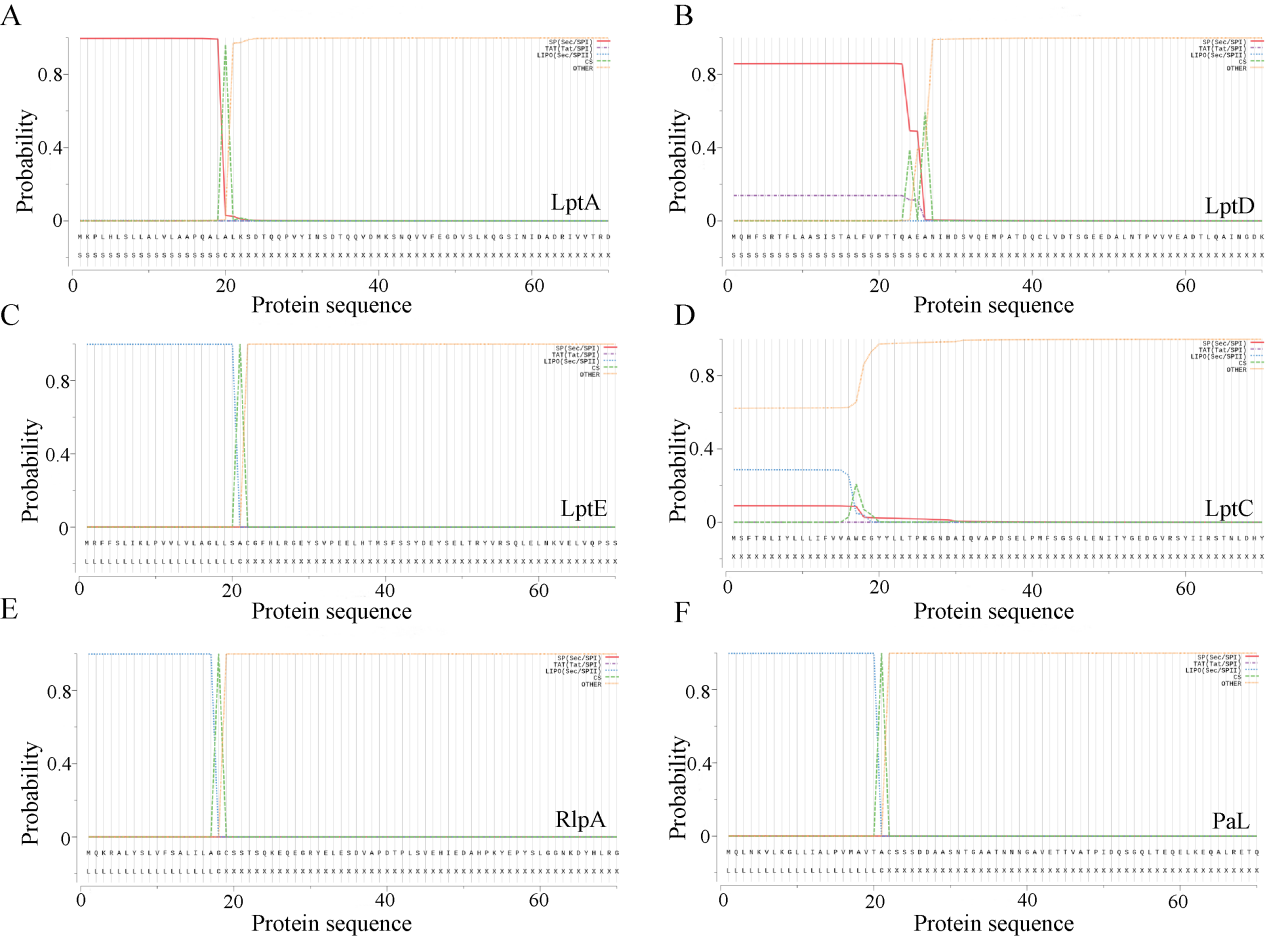
**

**Supplementary figure 1. Predictions of signal peptide by SignalP-5.0**

(A-D) The signal peptide prediction of LptA, LptD, LptE and LptC proteins related to LPS assembly. (E, F) The signal peptide prediction of RlpA, Pal proteins related to PGN assembly.

**
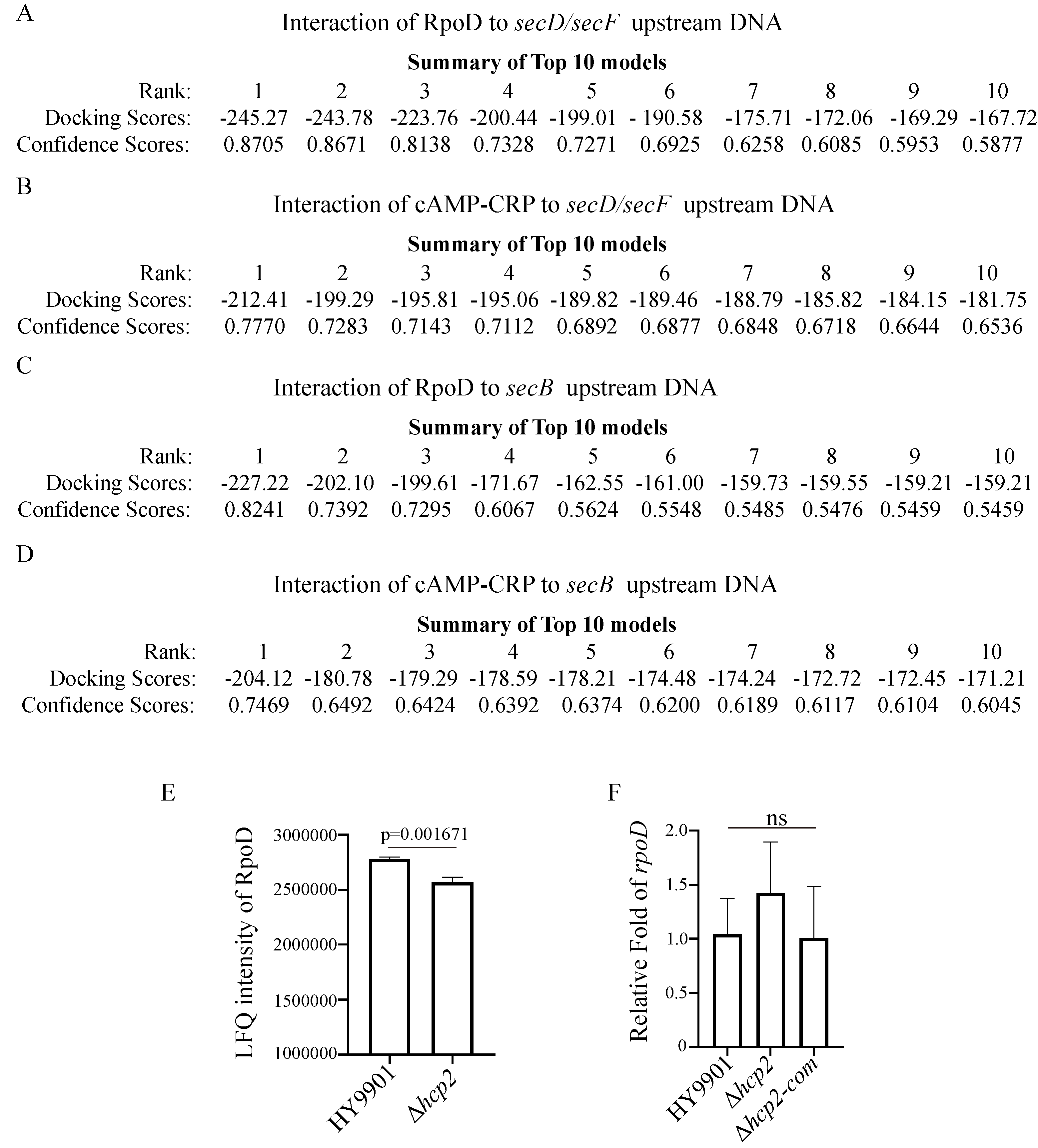
**

**Supplementary figure 2. The Summary of top ten molecular docking models and the expression regulation of *rpoD* in *V. alginolyticus***

1. B) The docking and confidence scores of top ten models of RpoD, cAMP-CRP interacting with the upstream DNA sequence of *secD/secF* genes. (C, D) The docking and confidence scores of top ten models of RpoD, cAMP-CRP interacting with the upstream DNA sequence of *secB* genes. (E, F) The expression regulation of *rpoD* gene by *hcp2*. HY9901-ev: the wild type strain carried with pBBR1-MCS1 empty vector; Δ*hcp2-*ev: the Δ*hcp2* strain carried with pBBR1-MCS1 empty vector; Δ*hcp2-*com: the Δ*hcp2* strain carried with pBBR1-MCS1-*hcp2* vector.

**Supplementary table 1. qPCR primers used in the study**

| Forward/Reverse primers | Primer sequence (5’-3’) |
| --- | --- |
| *tlr2*-qF  *tlr2*-qR | CTCGTCCCATCGGTTCAGTC  GCTCTTAGTGCCACCTTCCT |
| *tlr4*-qF  *tlr4*-qR | GGAATAATGGGCAGCCGTAAG  AGCGACACCAGGAACTATCAATG |
| *tlr5a*-qF  *tlr5a*-qR | TTCTGATGCTATTATTCATTTGG  AAAGCGAAGTAGCCATCAGTT |
| *tlr5b*-qF  *tlr5*b-qR | ATCGGCTATAATGCCATCTGC  CGAGTCCTGGTGTTTGTTGCT |
| *il6*-qF  *il6*-qR | TCCTCTCCTCAAACCTTCAGACCGC  TCTTTCCCTCTTTTCCTCCTGCTCA |
| *il10*-qF  *il10*-qR | ACTGACTGTTGCTCATTTGTG  TAGGGACTGTTTATGTTATGTTT |
| *il12a*-qF  *il12a*-qR | GAACTCCTACAAGCCCAGCAC  AGGGACACTCGGTCGTCAAAC |
| *il12ba*-qF  *il12ba*-qR | GTTGTTTTGTCTTTATTCGTTG  CATGTGATATTATGCCCTTCA |
| *il12bb*-qF  *il12bb*-qR | ACGATAAATGGACTGCTTGG  TCTTTGCTGCTGGATTGTTT |
| *tnfα*-qF  *tnfα*-qR | GCCTTTACCGCTGGTGATAGT  TGTTGATTGCCCTGGGTCTTA |
| *β-actin*-qF  *β-actin*-qR | CGTCTGGATCTAGCTGGTCGTGA  CAATTTCTCTTTCGGCTGTGGTG |
| *secB*-qF  *secB*-qR | AGCAGCACCTCAAGACGCACAAC  CAACTTCGTACACGCCTTCACCA |
| *secD*-qF  *secD*-qR | TCTTGGTCGTAACTTCCGTAT  TAGTAAAGCAGCGTAAATAGCA |
| *secF*-qF  *secF*-qR | TTCACTGGCGGTACTCTTATC  TTGGGACCTACGAACTCAATA |
| *rpoD*-qF  *rpoD*-qR | TCAACCAACTACGCAGCACCT  CTCGCGGAATACGTCTAGCAT |
| *16SrRNA*-qF  *16SrRNA*-qR | TTGCGAGAGTGAGCGAATCC |
|  | ATGGTGTGACGGGCGGTGTG |
